# Insight into cytotoxic and molecular mechanisms of bee venom and its nanoemulsion against West Nile virus in vitro

**DOI:** 10.1038/s41598-026-60587-7

**Published:** 2026-07-14

**Authors:** Abeer M. Salem, Heba Seyam, Sameh Ismail, Mohammed A. Abd El-Mobdy, Eman E. Zaher, El-Sayed H. Shaurub, Soad A. ElKenawi

**Affiliations:** 1 Department of Biotechnology, Faculty of Science, Cairo University, P.O. Box 12613, Giza, Egypt; 2 Honey Bee Research Department, Agricultural Research Center, Plant Protection Research Institute, Dokki, Giza, Egypt; 3 Faculty of Nanotechnology for Postgraduate Studies, Cairo University, Sheikh Zayed Branch, P.O. Box 12588, Sheikh Zayed City, Giza Egypt; 4 Department of Zoology, Faculty of Science, Zagazig University, Zagazig, Egypt; 5 Department of Entomology, Faculty of Science, Cairo University, B.O. Box 12613, Giza, Egypt

**Keywords:** Bee venom, Bee venom nanoemulsion, Antiviral, West Nile virus, Vero cell cytotoxicity, RT-qPCR, Molecular docking, Biotechnology, Diseases, Drug discovery, Microbiology

## Abstract

West Nile virus (WNV) causes West Nile fever and is primarily transmitted by *Culex* spp. mosquitoes. The primary hosts of WNV are birds, while humans and horses can also develop illness following infection. Currently, no specific antiviral therapy exists for WNV, and vaccination remains the only effective preventive measure. This study aimed to evaluate the efficiency of the crude bee venom (BV) and its nanoemulsion (NE) on WNV by determining in vitro Vero cells cytotoxicity and viral titer by RT-qPCR. We also conducted BV peptides-envelope glycoprotein of WNV interactions by molecular docking analysis. The NE of BV was characterized by dynamic light scattering (DLS) and zeta potential analyses. The DLS revealed that the size of NE was greater than that of BV. Also, the DLS showed that the polydispersity index of NE was less than that of BV (0.349 and 0.557, respectively). Zeta potential analysis of NE was slightly less than that of BV (23.0 and 23.7 mV, respectively). Both treatments showed low cytopathic effects on Vero cells compared to positive control infected with WNV. RT-qPCR revealed that the titer of WNV was lower in cells treated with BV and its NE than in controls. Molecular docking revealed BV peptides-envelope glycoprotein of WNV interactions, with binding energy of − 5.2, − 4.5, − 13.0, − 10.8, and − 5.1 kcal/mol for the peptides apamin, tertiapin, melittin, mast cell degranulating peptide, and secapin-2 peptide, respectively. BV-NE demonstrated preliminary in vitro anti-WNV activity requiring mechanistic, toxicological, and in vivo validation.

## Introduction

West Nile virus (WNV) is a positive-stranded RNA virus that causes West Nile fever. It is an arthropod-borne virus belonging to the Flaviviridae family, genus Flavivirus, and is part of the Japanese encephalitis serocomplex. In nature, WNV is maintained in a transmission cycle primarily involving Culex mosquitoes and birds; horses and humans serve as incidental (dead-end) hosts^1,2^. Although many bird and mammal species can become infected with WNV, severe clinical symptoms as disorientation, paralysis, coma, and even death; are typically observed only in infected humans and horses. Huhn et al.^3^. WNV is transmitted to humans by the bite of infected mosquitoes, which become carriers after feeding on viremic vertebrate hosts, primarily birds^4^. There is currently no licensed human vaccine for WNV. Prevention for humans relies on mosquito control, while effective, authorized vaccines are available for horses^5^. Most established methods for assessing viral neutralization evaluate the inhibition of viral entry by measuring the reduction in plaque formation in monolayers of susceptible cell lines. Li^4^. For preliminary characterization and evaluation of the pathogenesis and virulence of a (novel) WNV strain, both in vitro and in vivo systems have been employed, including the infection of various cell lines. Lim^6^, Szentpáli-Gavallér^7^ and primary cell cultures^8,9^. WNV replication can be studied in vitro using various cell lines derived from different sources. Common cell lines used include C6/36 (mosquito cells), Vero cells (African green monkey kidney cells) and BHK-21 (baby hamster kidney). These cell lines are utilized for viral isolation, propagation, attenuation, and a wide range of molecular investigations. Ben-Nathan et al.^10^, Lim^6^, Visser^11^.

Apitherapy, the therapeutic use of bee-derived products such as pollen, honey, propolis, and bee venom (BV), has been practiced for thousands of years and was particularly prominent in ancient Egyptian and Greek medicine^12^, Azam et al.^52^. Bee venom (BV) is a rich source of bioactive molecules, including peptides, amines, enzymes, free amino acids, and low-weight compounds^13,14^. According to Yaacoub et al.^15^, the complete chemical makeup of BV remains incompletely characterized. Nevertheless, some identified small-molecule constituents include carbohydrates, alcohols, and polyols (such as pantothenic acid and quinic acid), along with amines like histamine and phenylethylamine. Modified amino acids such as N-acetyl glutamic acid, N-acetyl alanine, N-acetyl aspartic acid, and N-methyl aspartic acid have also been reported. In addition, BV contains several peptides in lower abundance, including apamin, mast cell-degranulating peptide (MCD), adolapine, secapin, and procamine^16^. The dominant peptide, however, is melittin which is a 26-amino-acid sequence comprising roughly 40–60% of BV’s dry mass^14^. BV has been evaluated against several viruses within the Flaviviridae family, including flaviviruses such as WNV and hepatitis viruses such as Hepatitis C virus (HCV). Studies have shown that BV exerts direct antiviral effects on HCV, demonstrated by a marked reduction in infectious viral particles in cell culture^17^.

Nanoemulsions (NEs) are uniform dispersions composed of two immiscible liquids, typically characterized by droplet sizes below 100 nm. They can be formulated as either water-in-oil (W/O) or oil-in-water (O/W) systems^18,19^. NEs serve as versatile drug-delivery vehicles, offering several advantages, including the ability to incorporate both hydrophilic and hydrophobic substances in a single formulation, enhanced bioavailability and drug loading, controlled release, and protection of active compounds from enzymatic degradation^20,21^. Previous studies revealed that BV encapsulated in NEs enhances its delivery through the skin and may improve its anti-inflammatory and anti-rheumatic effects^22,23^.Topical delivery of BV-NEs has shown promise in reducing inflammation in animal models of arthritis, potentially due to BV’s ability to modulate pro-inflammatory pathways^24^. Aher et al.^51^ recently noted that nanomaterials can enhance the therapeutic efficacy of BV by enabling targeted delivery and facilitating its reach to specific cell populations.

By using recent and advanced techniques and programs, the protein–protein docking simulations have been employed to examine the antiviral potential of honey bee venom peptides by predicting their interactions with viral targets^25^, and to assess the binding affinity of selected phytocompounds against WNV proteins^26^. Accordingly, the present study aimed to assess the effectiveness of crude bee venom and its nanoemulsion against West Nile virus (WNV) by evaluating cytotoxicity in Vero cells and quantifying viral titers using RT-qPCR. In addition, molecular docking was performed to analyze the interactions between bee venom peptides and the WNV envelope glycoprotein.

## Materials and methods

### Honey bee venom (HBV) extraction by the electrical stimulated method

Honey bee workers were collected from the apiary of the Honey Bee Research Department, Agricultural Research Center, Dokki, Giza, Egypt (30.0379°N, 31.2083°E). Bees were maintained under standardized feeding conditions using 50% sugar syrup (1:1 sucrose). A single pooled batch (1 g) of crude bee venom was collected during the summer season from approximately 10,000 worker honey bees using the electrostimulation method described by Benton et al.,^27^, with a slight modification. The same pooled batch of bee venom was used throughout the study to minimize batch-to-batch variability. The bee workers stung the glass plates with plastic foil during the electro-stimulation. After drying for 35 min at 27 °C and 50% relative humidity, dried HBV (powder) was scraped and collected from the venom collector’s glass slide with a sharp scraper. HBV powder was then kept frozen at − 20 °C until use.

### Preparation of honey bee venom nanoemulsion

A stock of bee venom (BV) dissolved in deionized water was prepared to obtain BV 5 mg/ml concentration. Then, Tween 80, which acts as an emulsifier and a non-ionic surfactant, was added to the solution and mixed well using a magnetic stirrer.Then, garlic oil as the oil phase was added, mixed thoroughly, and centrifuged at1000 rpm for 5 min at room temperature according to Yousefpoor et al.^23^.

### Physical properties analyses

#### Dynamic light scattering (DLS)

Particle size distribution and polydispersity index were determined using a dynamic light scattering analyzer (MICROTRAC Nano WAVE II, UK) equipped with a 4 mW He-Ne laser (λ = 633 nm). Analyses were conducted at 25 °C with a 90° scattering angle. Prior to measurement, samples were diluted with ultrapure DMSO and passed through a 0.22 μm membrane filter^23,28^.

#### Zeta potential analysis

Surface charge characteristics were assessed using a Malvern Zetasizer Nano ZS instrument. Measurements were carried out at 25 °C in folded capillary cells (DTS1070). Zeta potential values were calculated from electrophoretic mobility using Smoluchowski’s equation. pH-dependent assessments were performed across a range of 4–10, with pH adjustments made using 0.1 M HCl and 0.1 M NaOH^28,29^.

#### Virus propagation

West Nile virus (WNV) (Eg101 strain, GenBank accession # AF260968) was propagated in African green monkey Vero cell line. The virus strain and Vero cell line were obtained from Tissue Culture laboratory in VACSERA organization.

#### Cell culture preparation

Vero cells were maintained in DMEM supplemented with 10% FBS, glutamine (2 mM), penicillin (100 µg/ml), and streptomycin (100 µg/ml). The cells were cultured at 37 °C in a humidified incubator with 5% CO₂. The WNV strain and Vero cell line used in this study were obtained from the Tissue Culture Laboratory, VACSERA, Egypt. All experiments involving live WNV and infected Vero cell cultures were conducted under Biosafety Level 3 (BSL-3) containment conditions in accordance with institutional biosafety regulations and VACSERA laboratory safety procedures. Viral manipulations and cell culture procedures were performed in a certified biological safety cabinet using appropriate personal protective equipment and standard virological containment practices.

#### Cytotoxicity assay

To study the effects of crude honey bee venom and its nanoemulsion on the viability and proliferation of Vero cells, the MTT assay was performed using 2-fold serial dilutions ranging from 100 µg/ml to 0.01 µg/ml according to Hansen et al.^30^ and Van de Loosdrecht et al.^31^. This concentration range was selected to evaluate concentration-dependent cytotoxic effects and determine the non-cytotoxic concentrations and IC₅₀ values of the tested formulations. For antiviral evaluation, crude bee venom and bee venom nanoemulsion were each tested at their respective maximum non-cytotoxic concentrations, as determined by the MTT assay. Thus, the comparison between formulations was based on their maximum biologically safe concentrations rather than on equivalent bee venom doses. Vero cells were seeded in 96-well tissue culture plates (Nunc™, Thermo Fisher Scientific) at 100 µl complete culture medium per well and incubated at 37 °C for 24 h. An inverted microscope was used to study and record morphological changes (Helmut HundGmbH, Wetzlar, Germany). After 24 h of cell incubation, the culture media was removed, and cells were washed twice with sterile PBS prior to the addition of the reagent MTT. Then, 25 µl of MTT solution (5 mg/ml Cat. No. M6494) was added to each well and the plates were incubated for an additional 4 h. The supernatant was then gently removed, and 150 µl of dimethyl sulfoxide (DMSO) was added to dissolve the formazan crystals. Optical density (OD) was measured at 540 nm using a LERX800 Biotek-USA ELISA reader. Cell viability (%) was calculated using the formula: Viability (%) = (OD of treated cells / OD of untreated cells [negative control]) × 100.

The half-maximal inhibitory concentration (IC50) values were determined by fitting the survival curves using MasterPlex 2010 software, Version 2.0.77 (MiraiBio, Hitachi Solutions America, Ltd.; https://masterplex-2010.software.informer.com/). Chemical structures and molecular modeling analyses were performed using ChemOffice 3D Bio, Version 15.0.0.106 (PerkinElmer Informatics; https://perkinelmerinformatics.com/products/research/chemdraw/). The IC_50_ can be calculated using the following formula:

Log_10_50% end point = log_10_ dilution above 50% - (Mortality above 50%)/(Mortality above 50% - Mortality below 50%).

### Effect of crude honey bee venom and nanoemulsion bee venom against West Nile Virus

Antiviral assays of crude bee venom and its nanoemulsion were conducted to evaluate their effects on WNV-induced cytopathic effects (CPE) and viral titer reduction in infected Vero cells using 2-fold serial dilutions ranging from 100 µg/ml to 0.01 µg/ml. Prior to antiviral testing, cytotoxicity against Vero cells was assessed to determine experimentally defined non-cytotoxic concentrations for each formulation. Antiviral activity was subsequently evaluated using concentrations that did not produce detectable cytotoxic effects on Vero cells. Under these conditions, the bee venom nanoemulsion demonstrated antiviral efficacy at lower non-cytotoxic concentrations compared with crude bee venom. The selected concentrations were added to infected cell cultures and compared with untreated infected controls. The non-toxic concentrations of each treatment in D-MEM growth medium were prepared, and 0.1 ml of each dilution was added per well to Vero cell monolayers, either treated or left untreated. Uninfected wells served as negative controls, whereas infected, untreated wells served as positive controls. Plates were incubated at 37 °C and monitored daily using an inverted microscope. After three days, virus titers in treated and untreated cells were determined based on the endpoint cytopathic effect (CPE) assay following the method of Reed and Muench^32^. This method was used because antiviral activity was evaluated based on endpoint CPE observations in Vero cells. The key criterion for the definition of CPE is the morphological changes in Vero cells. A commonly used, strict selection criterion for “hits” in screening is > 80% cell viability at the highest concentration tested, allowing for the calculation of an accurate Selectivity Index. To evaluate the potential contribution of nanoemulsion components to antiviral activity and cytotoxicity, additional control groups including Tween 80 alone, garlic oil alone, and blank nanoemulsion (without bee venom) were included and analyzed under the same experimental conditions.

### Virus quantification

#### RNA extraction

RNA was extracted from clear cell line from the wells (negative control without infected virus and treatments) untreated wells (infected with WNV as a positive control) and from wells treated with the non-toxic concentrations of each sample using BIOZOLBIOFLUX (Catalog No. 10760055-1), following the manufacturer’s protocol. RNA quality and quantity were assessed by 1.5% agarose gel electrophoresis and a NanoDrop 2000 spectrophotometer (Thermo Scientific, USA). cDNA synthesis was subsequently carried out using the Thermo Scientific™ RevertAid™ First Strand cDNA Synthesis Kit (#K1621), with 0.40 µg of RNA used per reaction.

#### Oligonucleotide sequence of West Nile virus

Primer pairs targeting the env gene were designed based on the nucleotide sequence of the WNV reference strain Eg101 (GenBank accession number AF260968). The forward primer was WNV F: 5′-TGG ATT TGG TTC TCG AAG G-3′ (genome positions 1028–1046), and the reverse primer was WNV R: 5′-GGT CAG CAC GTT TGT CAT T-3′ (genome positions 1228–1210), as described by Shukla et al.^2^.

#### Real-time PCR (RT-qPCR)

Following the manufacturer’s instructions, RT-qPCR reactions were done using the Quanti-Tect SYBR^®^ Green PCR kit (Qiagen, Valencia, CA). PCR reactions were incubated at 95 °C for 2 min in the CFX96TM Real-time PCR Detection System using SsoFastTM Eva GreenVRsupermix (BioRad), followed by 40 cycles of 95 °C of denaturation for 15 s and annealing at 60 °C for 10 s and then 72 °C for 10 s. In a total of 25 µl for each reaction, 20 µM of primer and 2 µl of cDNA as a template were used in triplicate.

### Standard curve

A standard curve for RT-qPCR quantification was generated using cDNA obtained from WNV-infected untreated Vero cells (positive control). The concentration of the amplified cDNA was measured using a NanoDrop 2000 spectrophotometer (Thermo Scientific, USA). Ten-fold serial dilutions ranging from 7 × 10⁶ copies/mL to 7 × 10² copies/mL were prepared and used as reference standards for viral RNA quantification. RNA samples extracted from treated WNV-infected Vero cells were analyzed as unknown samples and quantified by comparison with the generated standard curve. Because the assay was designed for viral RNA quantification rather than host gene expression analysis, normalization to a housekeeping gene was not applied. Absolute viral RNA quantification was performed using a standard curve generated from ten-fold serial dilutions ranging from 7 × 10⁶ to 7 × 10² copies/mL. The RT-qPCR assay exhibited an amplification efficiency of 100.4% and a correlation coefficient (R²) of 0.998.

### In-silico study

#### Molecular docking study

The crystal structures of the West Nile virus envelope glycoprotein (PDB ID: 2i69) and the bee venom peptides Apamin (PDB ID: 7oxf), Tertiapin (PDB ID: 1ter), and Melittin (PDB ID: 2mw6) were retrieved from the Protein Data Bank (PDB). The structures of Mast Cell Degranulating Peptide (ID: P01499) and Secapin peptide (ID: 2-AF-I1VC85) were obtained from UniProt as Alpha Fold models. The 3D structures of the ligands were generated using ChemBio Office software and the DrugBank database. Protein preparation was carried out in UCSF Chimera by removing water molecules and co-crystallized ligands, adding polar hydrogens, and assigning partial charges. Molecular docking of ligands with the target proteins was conducted using Auto Dock Vina. Binding poses were generated and analyzed, binding energies were calculated, and key ligand–protein interactions were evaluated to determine binding affinity. Docking was performed with the following parameters: Num modes = 30, exhaustiveness = 32, energy range = 3, and a grid box encompassing the entire receptor with active torsions enabled.

**Table:** 

Ligands	Receptors
	West Nile virus envelope glycoprotein(PDB ID: 2i69)
Apamin (PDB ID: 7oxf),	49 active torsions
Tertiapin (PDB ID: 1ter)	64 active torsions
Melittin (PDB ID: 2mw6)	59 active torsions
Mast cell degranulating peptide (ID: P01499)	59 active torsions
Secapin peptide (ID: 2-AF-I1VC85)	50 active torsions

To ensure reliability, the molecular docking simulations were conducted in triplicate. From each simulation, the conformation exhibiting the lowest binding free energy was selected for further analysis. For every docking cycle, 30 structural models were generated, with the model displaying an RMSD of 0.000 retained as the optimal representative. Subsequent analyses focused on the evaluation of binding poses and calculation of binding energies to elucidate key molecular interactions between the ligands and their respective protein targets. The binding affinities of apamin, tertiapin, melittin, mast cell degranulating peptide, and secapinpeptide were systematically assessed for each protein. Post-docking, all ligand-protein complexes were subjected to energy minimization, consisting of 1000 steps of the steepest descent algorithm followed by 100 steps of the conjugate gradient method, using a minimization step size of 0.001 Å.

### Statistical analysis

All experiments, including cytotoxicity assays, antiviral assays, and RT-qPCR analyses, were performed in triplicate and repeated independently three times (*N* = 3 independent experiments). Data are presented as mean ± standard deviation (SD). Data normality was assessed using the Shapiro–Wilk test prior to one-way ANOVA. Differences among groups were analyzed using one-way ANOVA followed by Tukey’s Honestly Significant Difference (HSD) post hoc test for multiple pairwise comparisons, with adjusted *p* values (*p*adj < 0.05) considered statistically significant. Viral titers were analyzed and presented as log₁₀-transformed values; therefore, antiviral effects were interpreted based on direct log₁₀ titer reductions rather than percentage reductions. Cell viability (%) and RT-qPCR Ct values were analyzed using the same statistical framework.

## Results

### Physical properties analysis

#### Dynamic light scattering (DLS)

**Fig. 1:**
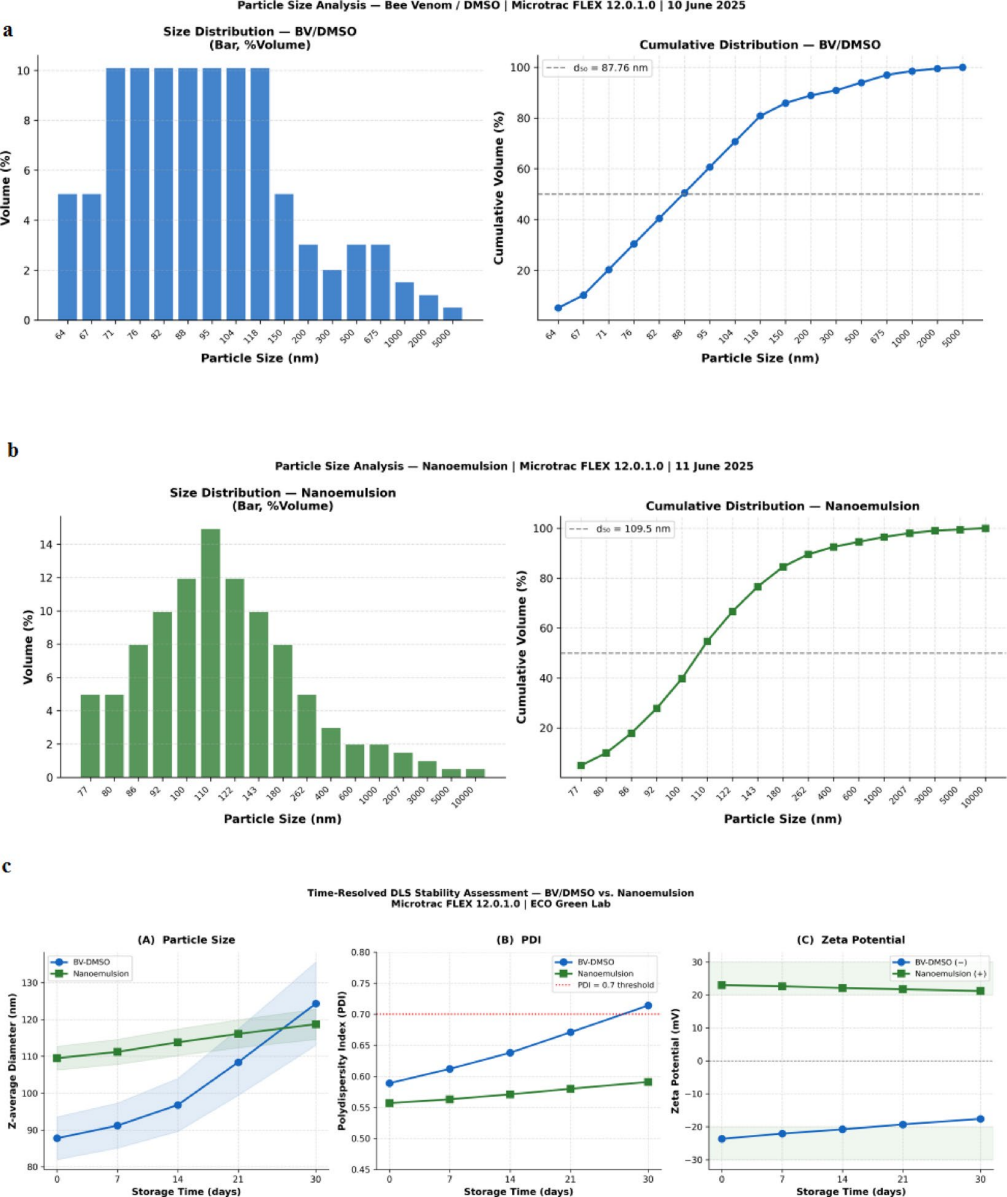
(**a**) Particle size distribution histogram and cumulative distribution curve of BV/DMSO. The left panel shows the particle size distribution as volume percentage per size channel, while the right panel presents the cumulative particle size distribution. (**b**) Particle size distribution histogram and cumulative distribution curve of the bee venom nanoemulsion. (**c**) Time-dependent dynamic light scattering (DLS) stability profiles of BV/DMSO and bee venom nanoemulsion during storage at 4 °C, including (**A**) Z-average particle size, (**B**) polydispersity index (PDI), and (**C**) zeta potential.

In addition to single-timepoint PDI measurements, a 30-day time-course stability study was performed during storage at 4 °C (*n* = 3 per time point). The BV/DMSO preparation exhibited a volume-weighted mean diameter (MV) of 675.0 nm, with a median diameter of 87.76 nm and a PDI of 0.589, indicating a moderately polydisperse system with a dominant population centered at 87.8 nm (100 vol%). In contrast, the nanoemulsion exhibited a median diameter of 109.5 nm, an MV of 2007 nm, and a PDI of 0.557. The broader MV reflects the wide tailing characteristic of polydisperse oil-in-water emulsions, whereas the dominant peak at 109.5 nm (100 vol%, width = 119.8 nm) indicates that the primary droplet population remained within the sub-200 nm range (Fig. 1a, b). During storage, the BV/DMSO preparation showed a progressive increase in particle size from 87.76 to 124.3 nm, accompanied by an increase in PDI from 0.589 to 0.714, indicating increased heterogeneity. In contrast, the nanoemulsion showed only a slight increase in size from 109.5 to 118.7 nm and a minimal change in PDI from 0.557 to 0.591, suggesting comparatively improved physical stability over the study period (Fig. 1c). The higher volume-weighted mean diameter compared with the median particle size reflects the polydisperse nature of nanoemulsion and the sensitivity of DLS to a small population of larger droplets or aggregates. Because larger particles scatter light much more strongly than smaller particles, they disproportionately influence the volume-weighted mean diameter. The minimal changes in median particle size (109.5–118.7 nm) and PDI during the 30-day stability study indicate that the dominant nanoemulsion population remained stable despite the presence of a minor fraction of larger particles.

#### Zeta potential analysis

**Fig. 2:**
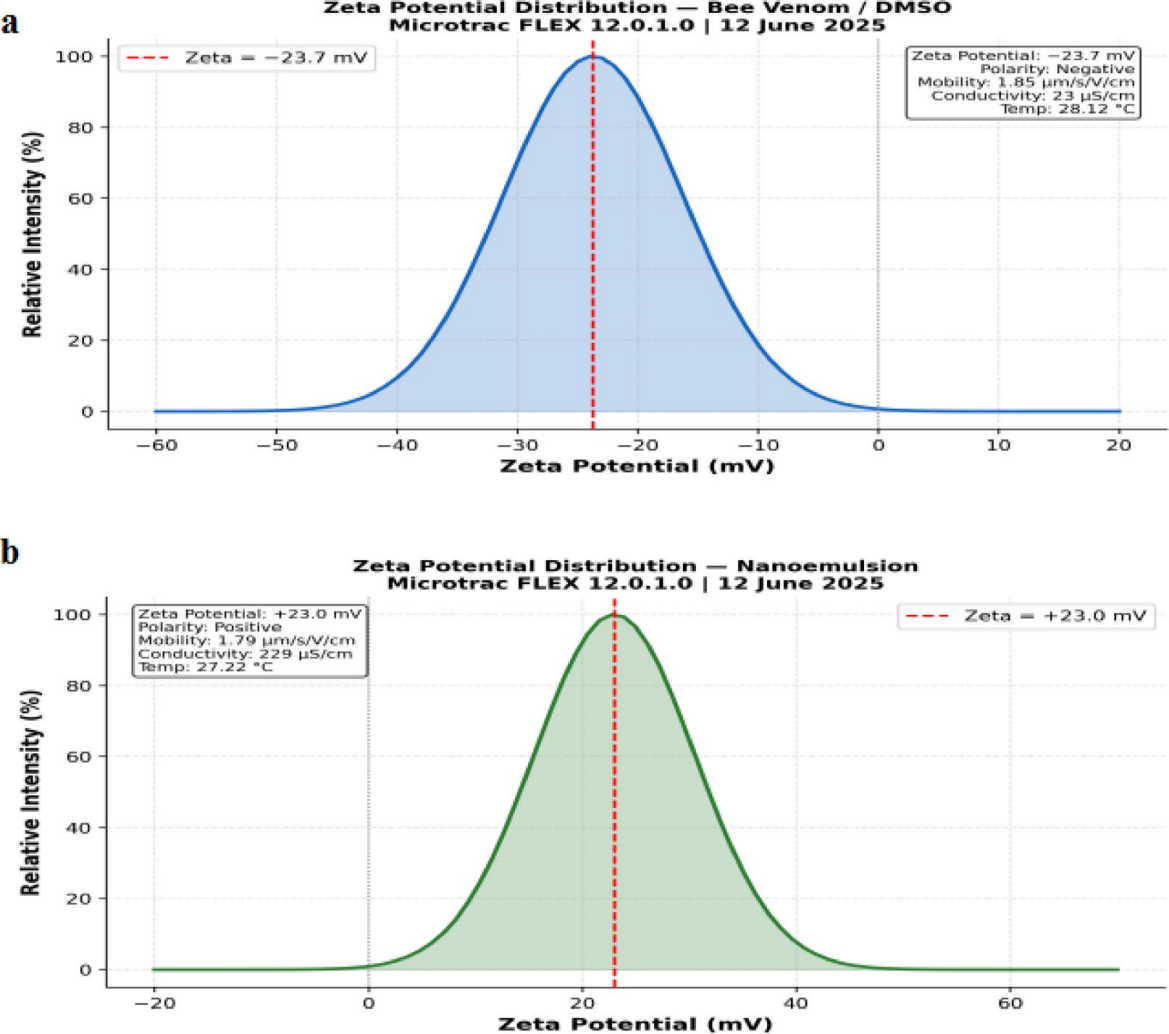
(**a**) Zeta potential distribution profile of BV/DMSO showing a negative surface charge with a peak value of − 23.7 mV. (**b**) Zeta potential distribution profile of the bee venom nanoemulsion showing a positive surface charge with a peak value of + 23.0 mV.

The BV/DMSO preparation exhibited a zeta potential of − 23.7 mV (negative polarity). The electrophoretic mobility was 1.85 μm/s/V/cm at a conductivity of 23 µS/cm, measured under an applied field strength of 10.0 kV/m. The measured value approaches but does not exceed the conventional stability threshold of ± 30 mV. The nanoemulsion exhibited a zeta potential of + 23.0 mV (positive polarity), with electrophoretic mobility of 1.79 μm/s/V/cm. The higher conductivity (229 µS/cm) compared to BV/DMSO reflects the ionic content of the emulsion formulation. Stability in nanoemulsions is supplemented by steric effects from the emulsifier, so zeta potential alone does not fully define colloidal stability in this system (Table 1; Fig. 2a, b).

**Table 1:** Zeta potential and electrokinetic parameters of crude bee venom and nanoemulsion bee venom.

Parameter	Crude bee venom	Nanoemulsion bee venom
Zeta potential (mV)	-23.7	+ 23.0
Polarity	Negative (Automatic)	Positive (Automatic)
Electrophoretic Mobility (uM/s/V/Cm)	1.85	1.79
Conductivity (uS/Cm)	23	229
Field Strength (Req/Act) (KV/m)	10/9.8	10/10.0

#### Cytotoxicity assay

**Fig. 3:**
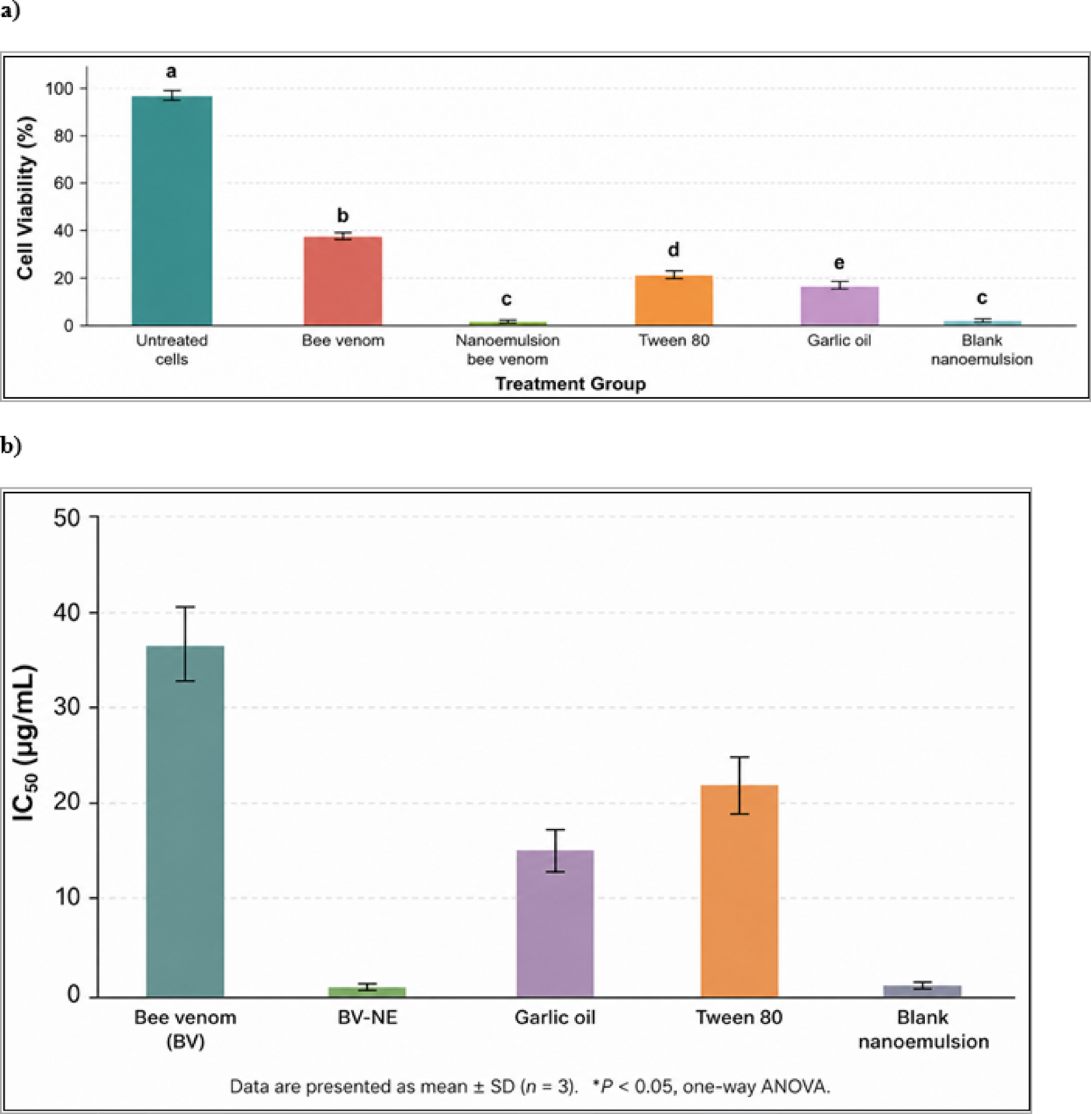
(**a**) Comparative cytotoxicity and cell viability (%) of cells across target treatments. Bars display mean cell viability (%) following exposure to Untreated cells, Bee venom, Nanoemulsion bee venom, Tween 80, Garlic oil, and Blank nanoemulsion. Error bars represent the Standard Error of the Mean ± SEM for *N* = 3 independent replicates. Lowercase letters (a–e) above the error bars indicate statistically significant groupings derived from Tukey’s Honestly Significant Difference (HSD) post-hoc test, using adjusted p-values (p_adj < 0.05) to correct for multiple comparisons. Bars sharing a common letter are not statistically different. (**b**) IC₅₀ values of crude bee venom (BV), bee venom nanoemulsion (BV-NE), garlic oil, Tween 80, and blank nanoemulsion against Vero cells.

Cytotoxicity and cell viability were assessed using an MTT assay. No cell death was observed in treated Vero cells at concentrations up to 10 µg/mL and 0.39 µg/mL for crude bee venom and bee venom nanoemulsion, respectively, after 24 h of incubation. The nanoemulsion formulation significantly reduced Vero cell viability compared with crude bee venom, indicating higher cytotoxicity, with IC₅₀ values of 0.94 µg/mL and 37.6 µg/mL, respectively (*p* < 0.05) (Fig. 3b). IC₅₀ values for garlic oil, Tween 80, and blank nanoemulsion were 15.2 µg/mL, 21.8 µg/mL, and 0.98 µg/mL, respectively. Cell viability differed significantly among treatment groups (one-way ANOVA, *p* < 0.05) (Fig. 3a). Untreated cells maintained the highest viability (97.67 ± 2.52%), whereas bee venom nanoemulsion and blank nanoemulsion reduced viability to near-zero levels (0.99 ± 0.09% and 1.58 ± 0.07%, respectively). Crude bee venom retained moderate viability (37.70 ± 0.26%), while Tween 80 and garlic oil exhibited intermediate cytotoxic effects, with viability values of 21.89 ± 0.02% and 16.43 ± 1.31%, respectively. Tukey’s HSD analysis demonstrated significant differences among most treatment groups, except between bee venom nanoemulsion and blank nanoemulsion.

Additional formulation controls, including garlic oil, Tween 80, and blank nanoemulsion, exhibited lower antiviral effects than the bee venom nanoemulsion formulation in both cytotoxicity and RT-qPCR analyses (Figs. 3b and 6).

**Fig. 4:**
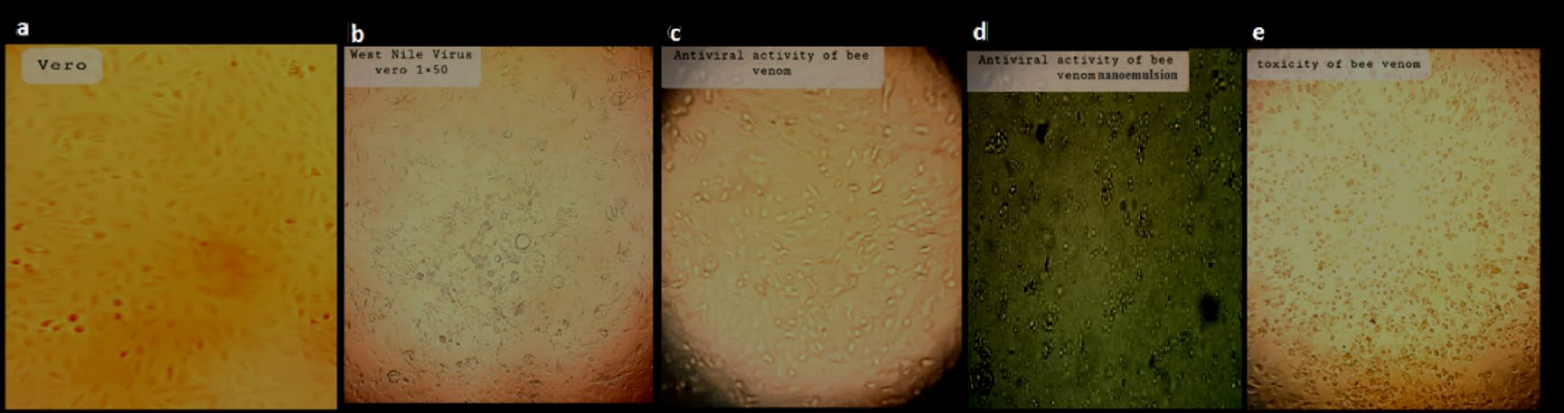
Representative micrographs of Vero cells under different treatment conditions. (**a**) Untreated control Vero cells exhibiting normal spindle-shaped morphology. (**b**) WNV-infected Vero cells showing pronounced cytopathic effects (CPE). (**c**,**d**) Protective antiviral effects of crude bee venom and bee venom nanoemulsion (BV-NE) against WNV infection in Vero cells. (**e**) Cytotoxic effect observed in treated Vero cells.

### Effect of crude bee venom and bee venom nanoemulsion on WNV viral titer in infected vero cells

**Fig. 5:**
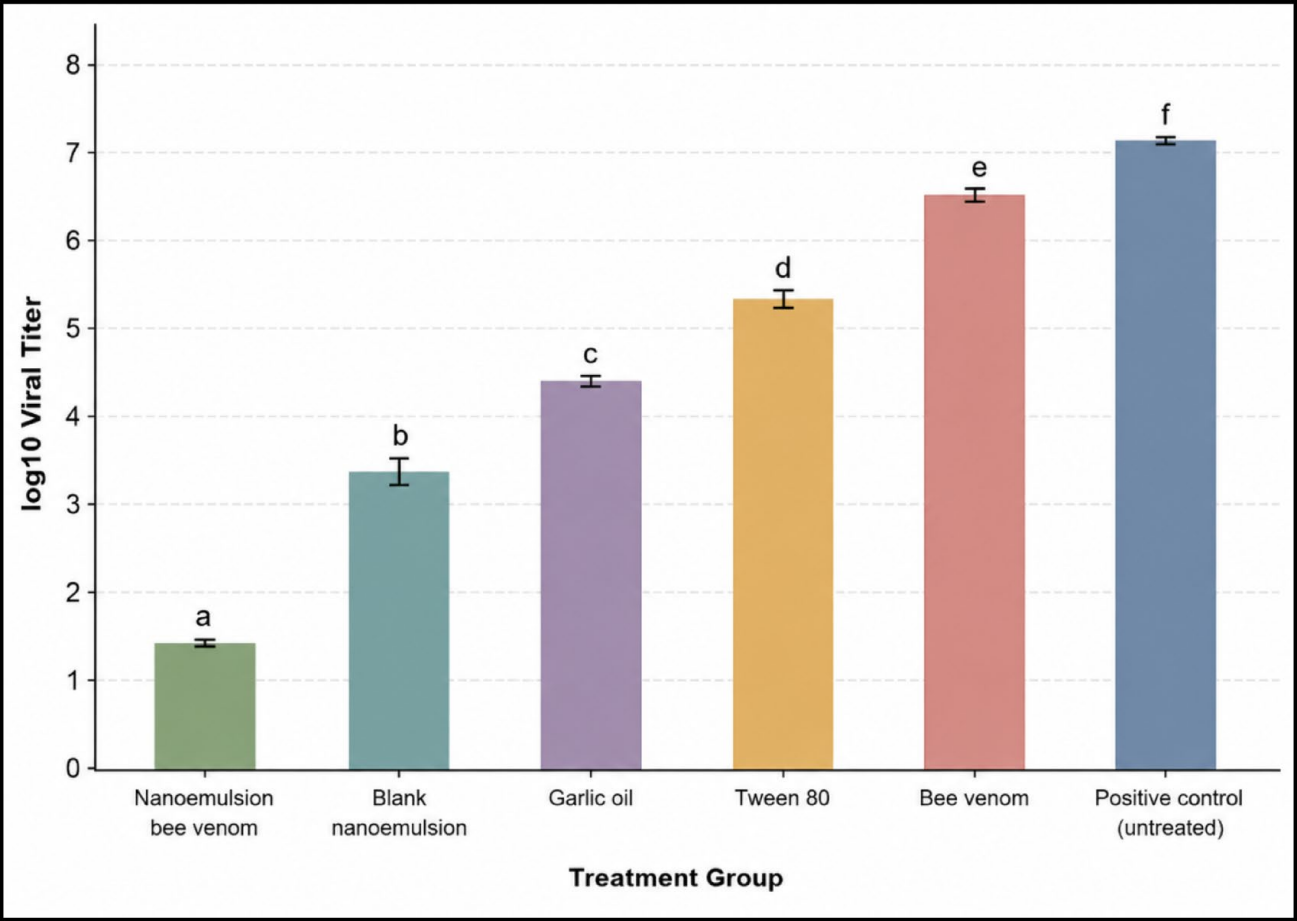
Effect of crude bee venom (BV) and bee venom nanoemulsion (BV-NE) on WNV viral titer in infected Vero cells after 3 days of incubation. Viral titers are expressed as log10 values. The positive control represents untreated WNV-infected cells. BV-NE treatment showed the greatest reduction in viral titer compared with crude BV and formulation control treatments. Data are presented as mean ± SEM (*n* = 3). Different lowercase letters (**a**–**f**) indicate statistically significant differences among groups (*p* < 0.05; one-way ANOVA followed by Tukey’s HSD multiple comparison test).

The cytopathic effect (CPE) in WNV-infected Vero cells was markedly reduced following treatment with crude bee venom (BV) and bee venom nanoemulsion (BV-NE) compared with the untreated infected control, which exhibited an initial viral titer of 7.15 log10 (Fig. 4). Both treatments demonstrated antiviral activity against WNV after 3 days of incubation in Vero cells. Treatment with a non-cytotoxic concentration of crude BV reduced the viral titer to 6.53 ± 0.12 log10, whereas BV-NE markedly reduced the viral titer to 1.45 ± 0.08 log10, indicating substantially greater antiviral activity at a lower non-cytotoxic concentration (Fig. 5).

Quantitative analysis demonstrated that BV-NE achieved the greatest antiviral effect, corresponding to a 5.70 log10 reduction relative to untreated cells (*p* < 0.05). Blank nanoemulsion, garlic oil, Tween 80, and crude bee venom showed progressively lower antiviral efficacy, with mean viral titers of 3.37 ± 0.47, 4.43 ± 0.12, 5.33 ± 0.21, and 6.53 ± 0.12 log10, respectively. Tukey’s HSD analysis confirmed significant differences among all treatment groups (*p*adj < 0.05) (Fig. 5). Although the blank nanoemulsion exhibited measurable antiviral activity, indicating that the formulation components contribute to viral inhibition, incorporation of bee venom into the nanoemulsion produced a significantly greater antiviral effect. Specifically, the bee venom nanoemulsion reduced the viral titer from 3.37 ± 0.47 log10 (blank nanoemulsion) to 1.45 ± 0.08 log10, representing an additional reduction of approximately 1.92 log10, with the difference being statistically significant (Tukey’s HSD, *padj* < 0.05). These findings demonstrate that incorporation of bee venom markedly enhanced the antiviral activity beyond that achieved by the carrier system alone, while the nanoemulsion served as both a delivery platform and a partially active antiviral formulation.

#### Real-time PCR (RT-qPCR)

**Fig. 6:**
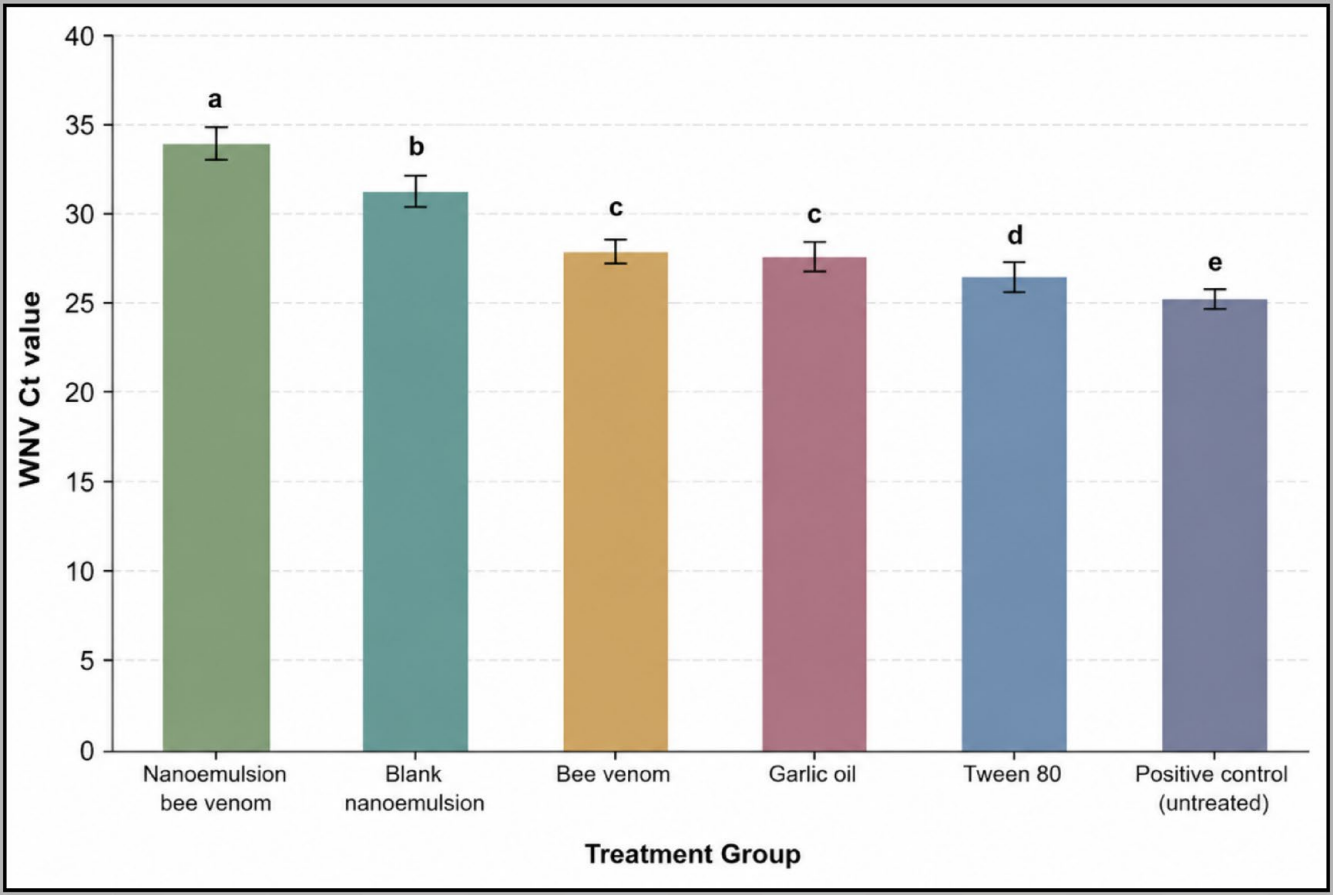
Effect of Tween 80, garlic oil, blank nanoemulsion, crude bee venom (BV), and bee venom nanoemulsion (BV-NE) on West Nile virus (WNV) Ct values determined by RT-qPCR in infected Vero cells. Higher Ct values indicate lower detectable viral RNA levels. Data are presented as mean ± SEM (*n* = 3). Different lowercase letters (**a**–**e**) indicate statistically significant differences among groups (*p* < 0.05; one-way ANOVA followed by Tukey’s HSD multiple comparison test).

RT-qPCR analysis demonstrated significant reductions in detectable WNV RNA levels following treatment with crude bee venom (BV) and bee venom nanoemulsion (BV-NE) compared with untreated infected cells. BV-NE yielded the highest mean Ct value (33.84 ± 0.52), indicating the greatest reduction in viral RNA, and differed significantly from all other treatment groups (Tukey’s HSD, *p*adj < 0.05). Blank nanoemulsion showed the second highest Ct value (31.31 ± 1.20). Tween 80 (28.31 ± 0.41) and crude bee venom (28.12 ± 0.46) exhibited statistically comparable effects, followed by garlic oil (27.59 ± 0.52). Untreated cells recorded the lowest Ct value (25.37 ± 0.44), consistent with uninhibited viral replication (Fig. 6). Absolute viral RNA quantification showed that untreated infected cells contained approximately 2.66 × 10⁵ RNA copies/mL. Treatment with the blank nanoemulsion reduced the viral RNA level to approximately 1.68 × 10² copies/mL, confirming that the nanoemulsion components possess intrinsic antiviral activity. Incorporation of bee venom into the nanoemulsion further reduced the viral RNA level to approximately 7.21 copies/mL, representing a substantially greater reduction than that achieved by the blank nanoemulsion alone. Bee venom alone, garlic oil, and Tween 80 yielded viral RNA levels of 5.91 × 10³, 1.71 × 10⁴, and 6.94 × 10³ copies/mL, respectively. These findings demonstrate that incorporation of bee venom markedly enhanced the antiviral activity beyond that achieved by the carrier system alone. The RT-qPCR assay showed excellent analytical performance, with an amplification efficiency of 100.4% and an R² value of 0.998.

#### Molecular docking analysis

**Fig. 7:**
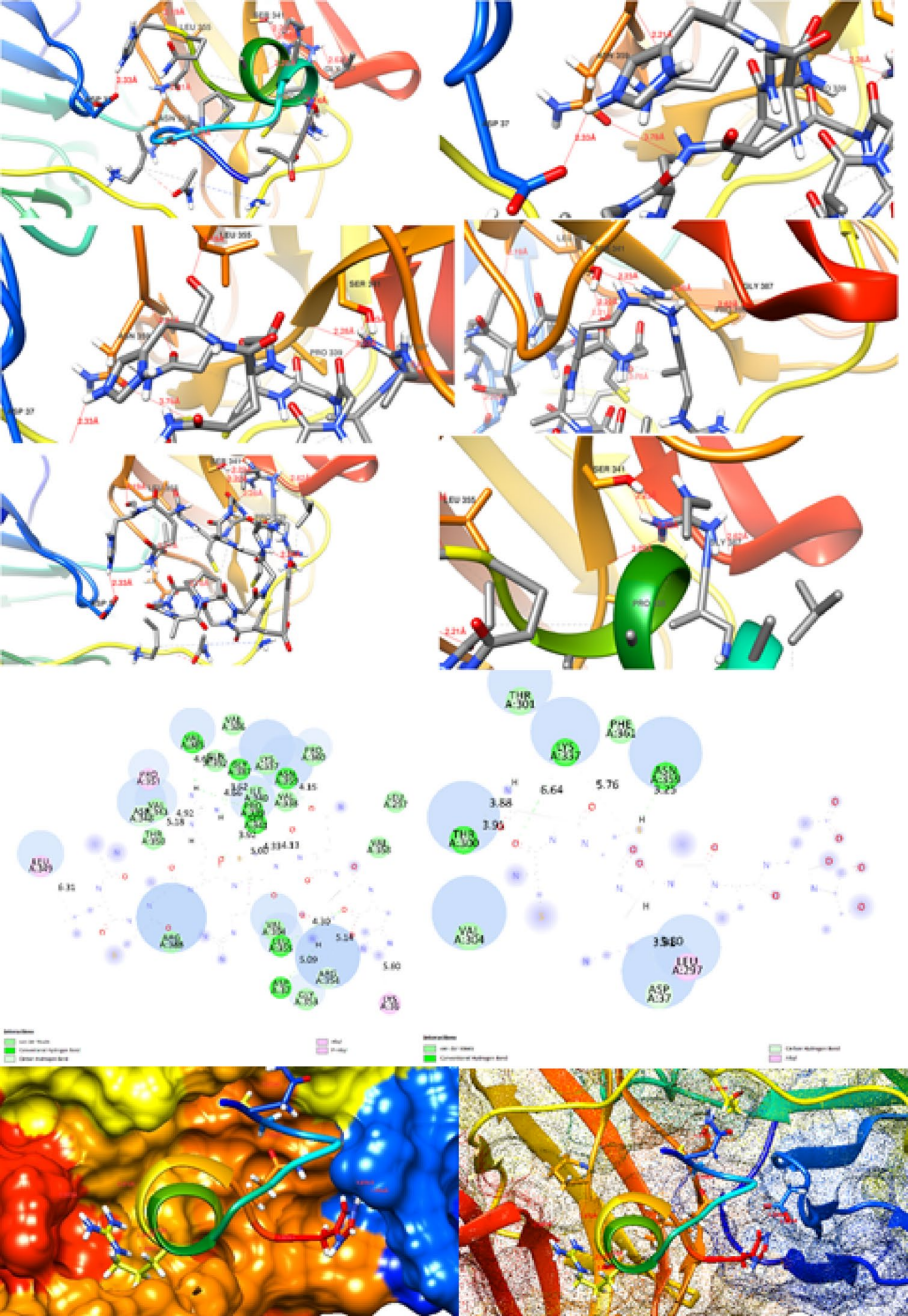
Representative 2D and 3D molecular docking models of Apamin bound to the West Nile virus (WNV) envelope glycoprotein. The predicted ligand–protein complexes demonstrate hydrogen bond formation and hydrophobic interactions between Apamin and key amino acid residues within the binding pocket of the WNV envelope protein. Apamin is represented in blue, whereas interacting residues are shown in orange. The calculated binding affinity of the Apamin–WNV complex was − 5.2 kcal/mol.

**Fig. 8:**
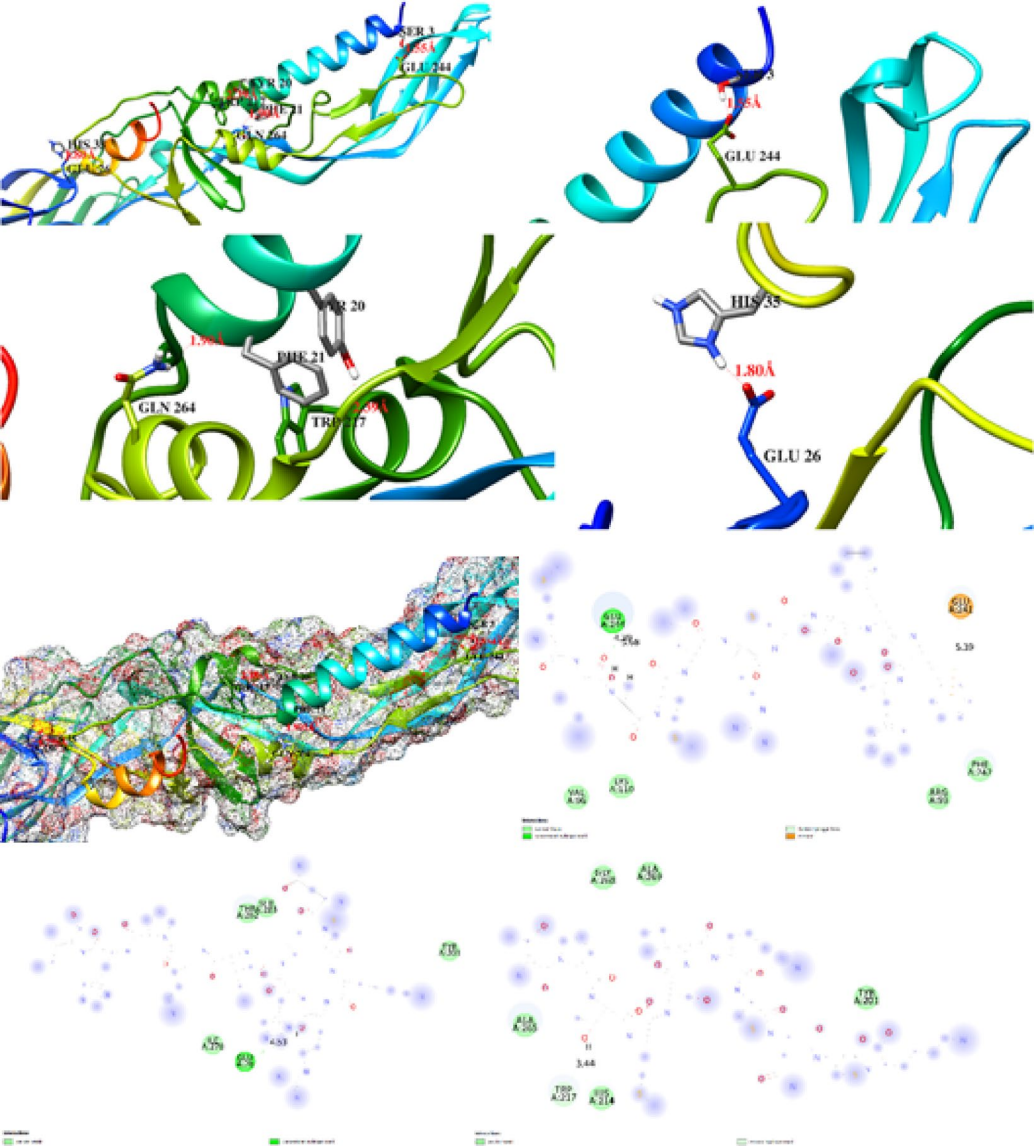

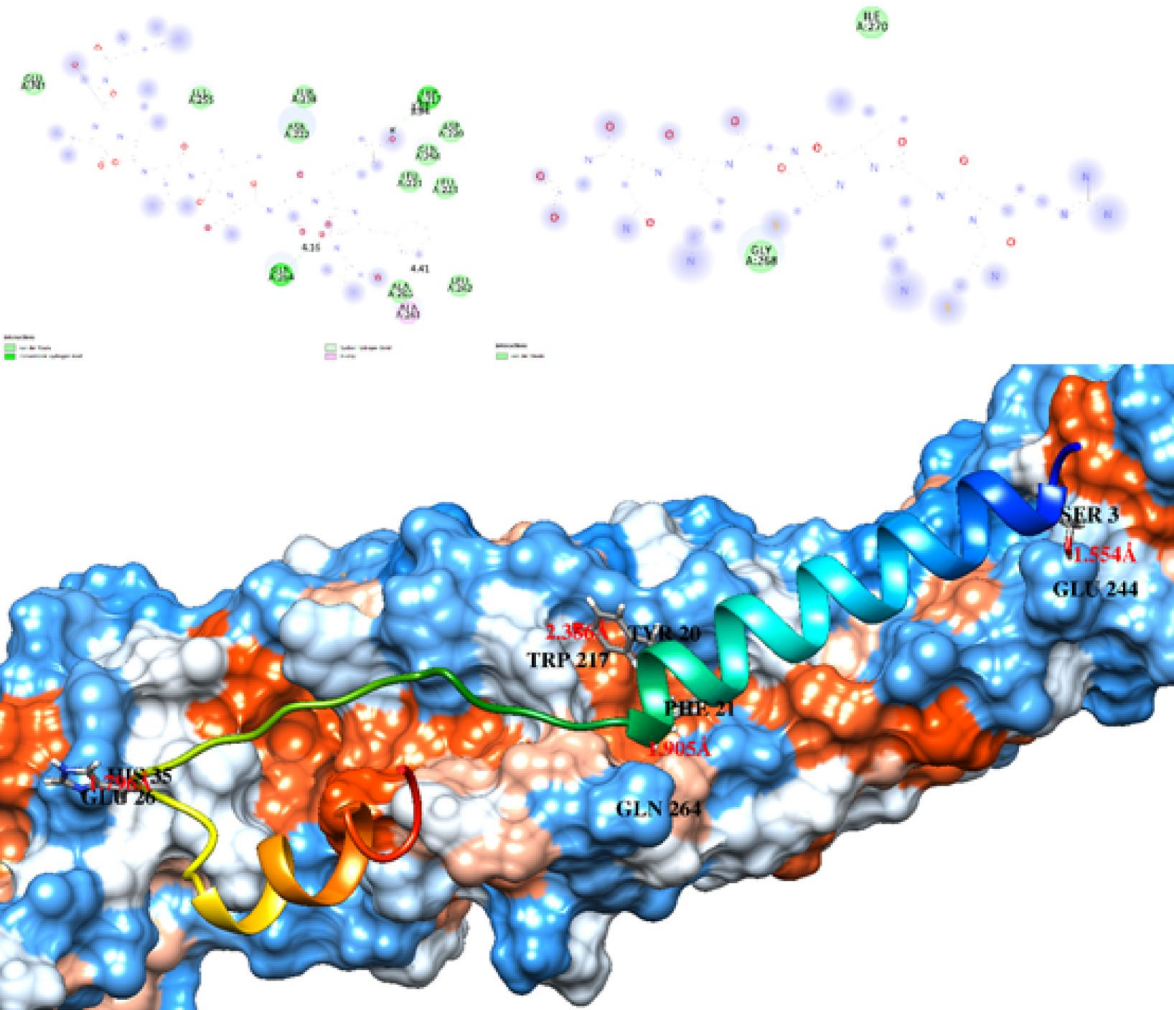
Representative 2D and 3D molecular docking models of mast cell degranulating peptide (MCD peptide) interaction with the West Nile virus (WNV) envelope glycoprotein. The predicted ligand–protein complexes demonstrate hydrogen bonding and hydrophobic interactions between the peptide and amino acid residues within the binding pocket of the WNV envelope protein. The mast cell degranulating peptide is represented in yellow, orange, and blue, while interacting residues of the WNV envelope glycoprotein are shown in gray and green. The calculated binding affinity of the peptide–WNV complex was − 10.8 kcal/mol.

**Fig. 9:**
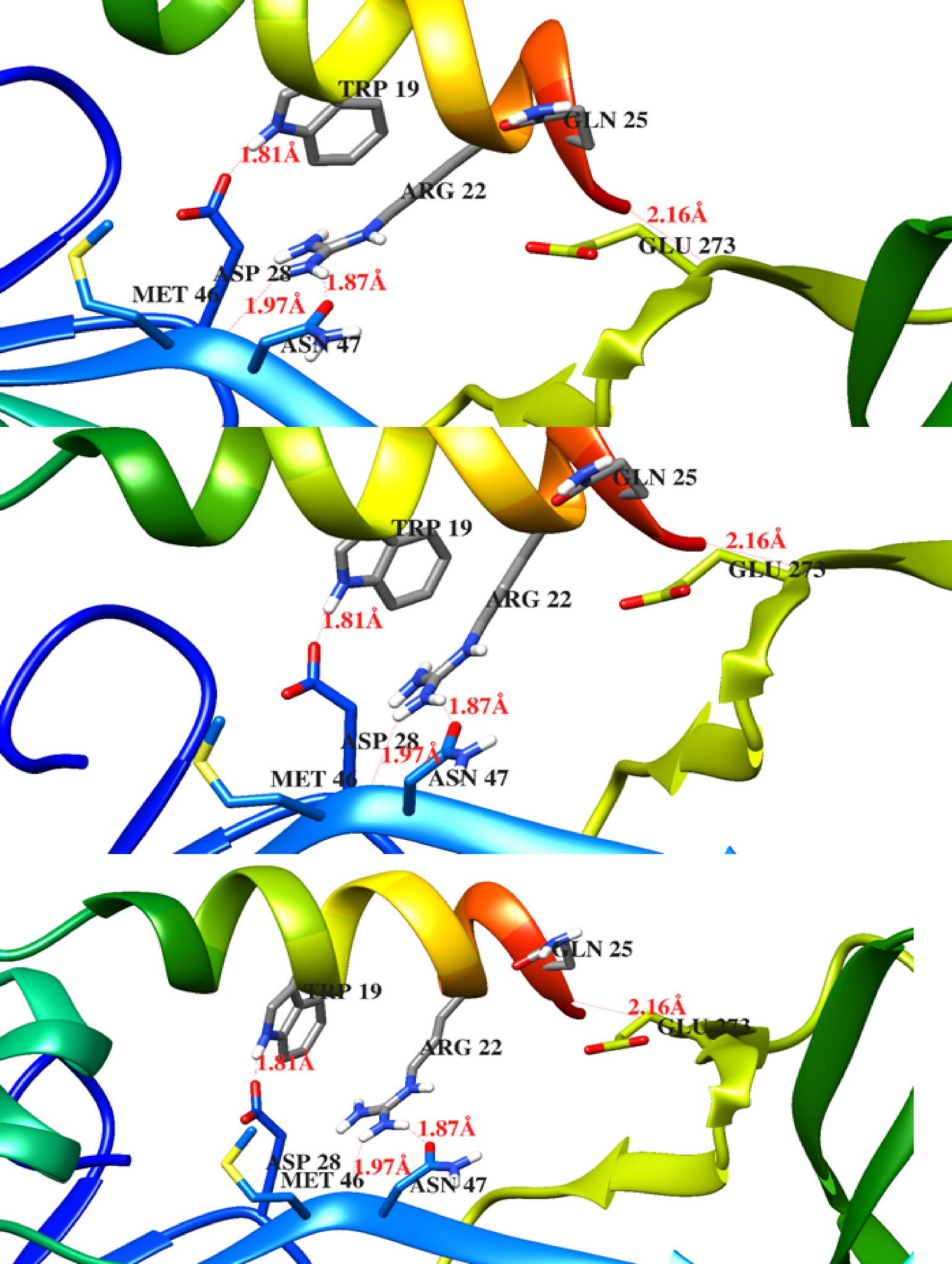

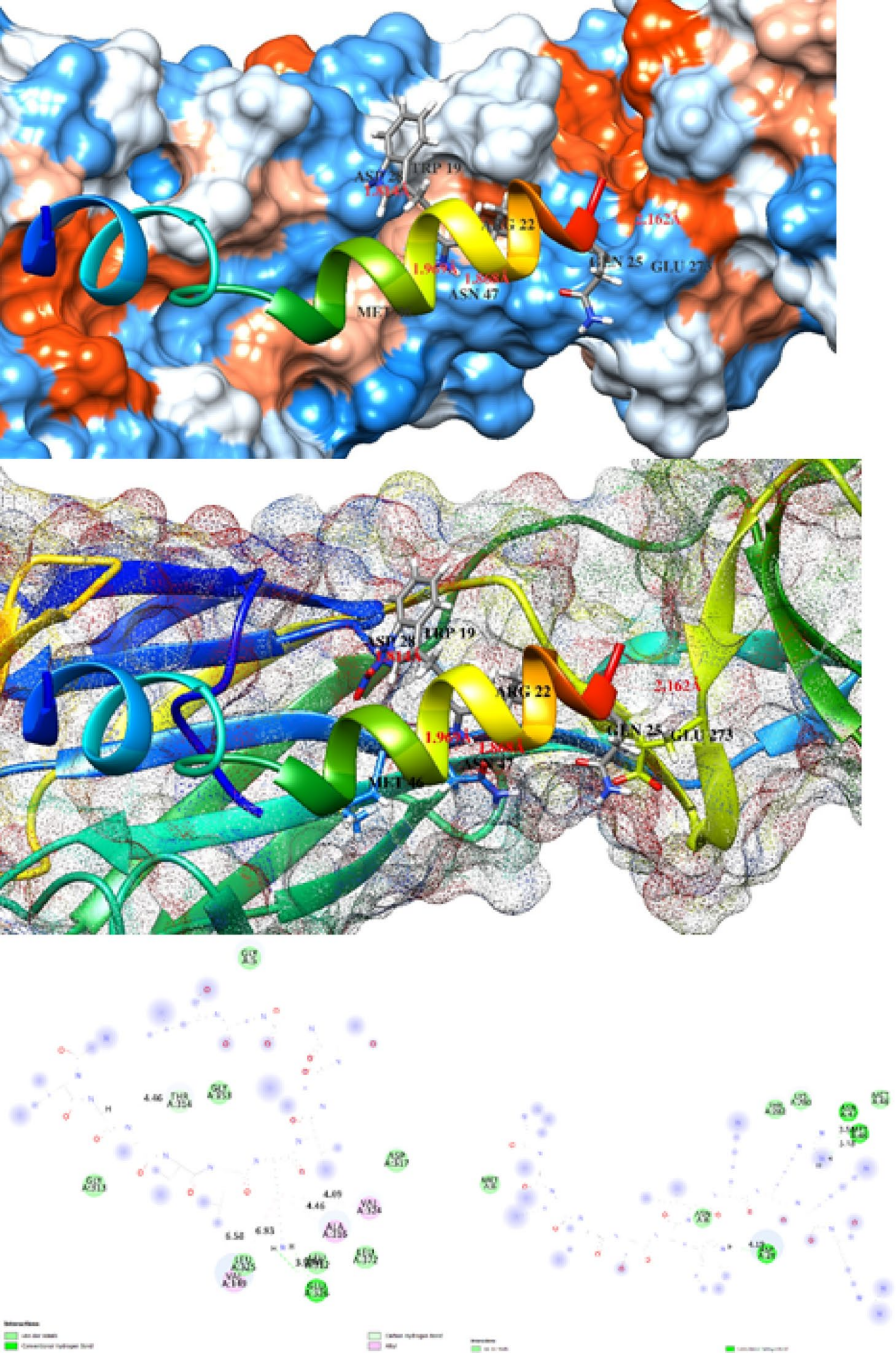
Representative 2D and 3D molecular docking models of melittin interaction with the West Nile virus (WNV) envelope glycoprotein. The predicted ligand–protein complexes illustrate hydrogen bonding and hydrophobic interactions between melittin and amino acid residues within the binding pocket of the WNV envelope protein. Melittin is represented in yellow and gray, while interacting residues of the WNV envelope glycoprotein are shown in blue and orange. The docking complex exhibited a binding free energy of − 13.8 kcal/mol.

**Fig. 10:**
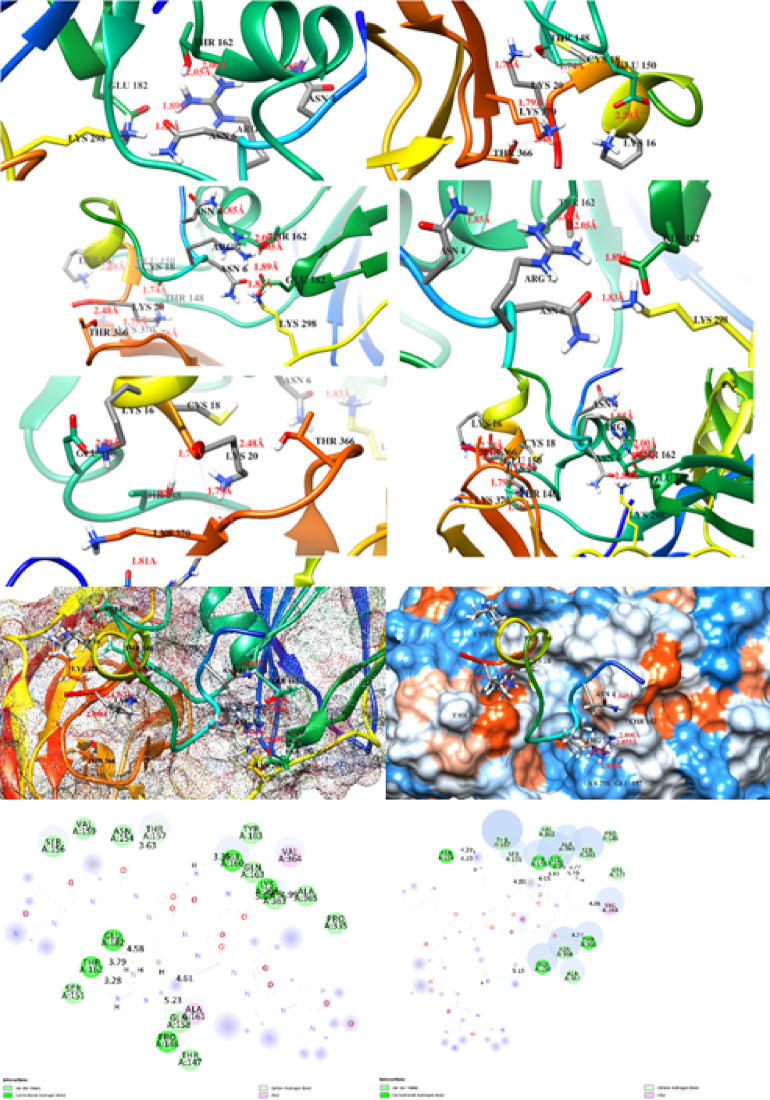
Representative 2D and 3D molecular docking models of tertiapin interaction with the West Nile virus (WNV) envelope glycoprotein. The predicted ligand–protein complexes illustrate hydrogen bonding and hydrophobic interactions between tertiapin and amino acid residues within the binding pocket of the WNV envelope protein. Tertiapin is represented in yellow, orange, and gray, while interacting residues of the WNV envelope glycoprotein are shown in blue and green. The docking complex exhibited a binding free energy of − 4.5 kcal/mol.

**Fig. 11:**
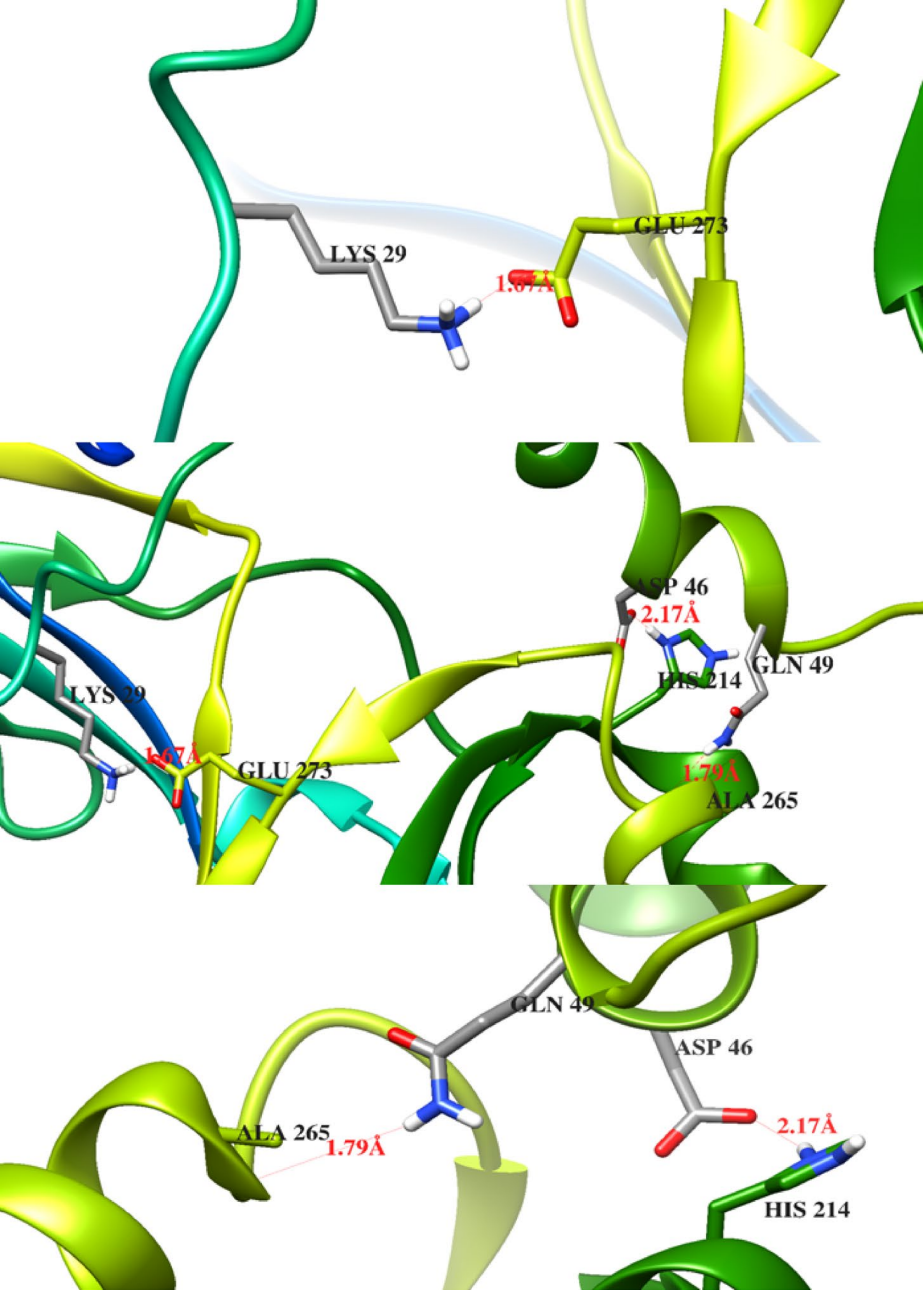

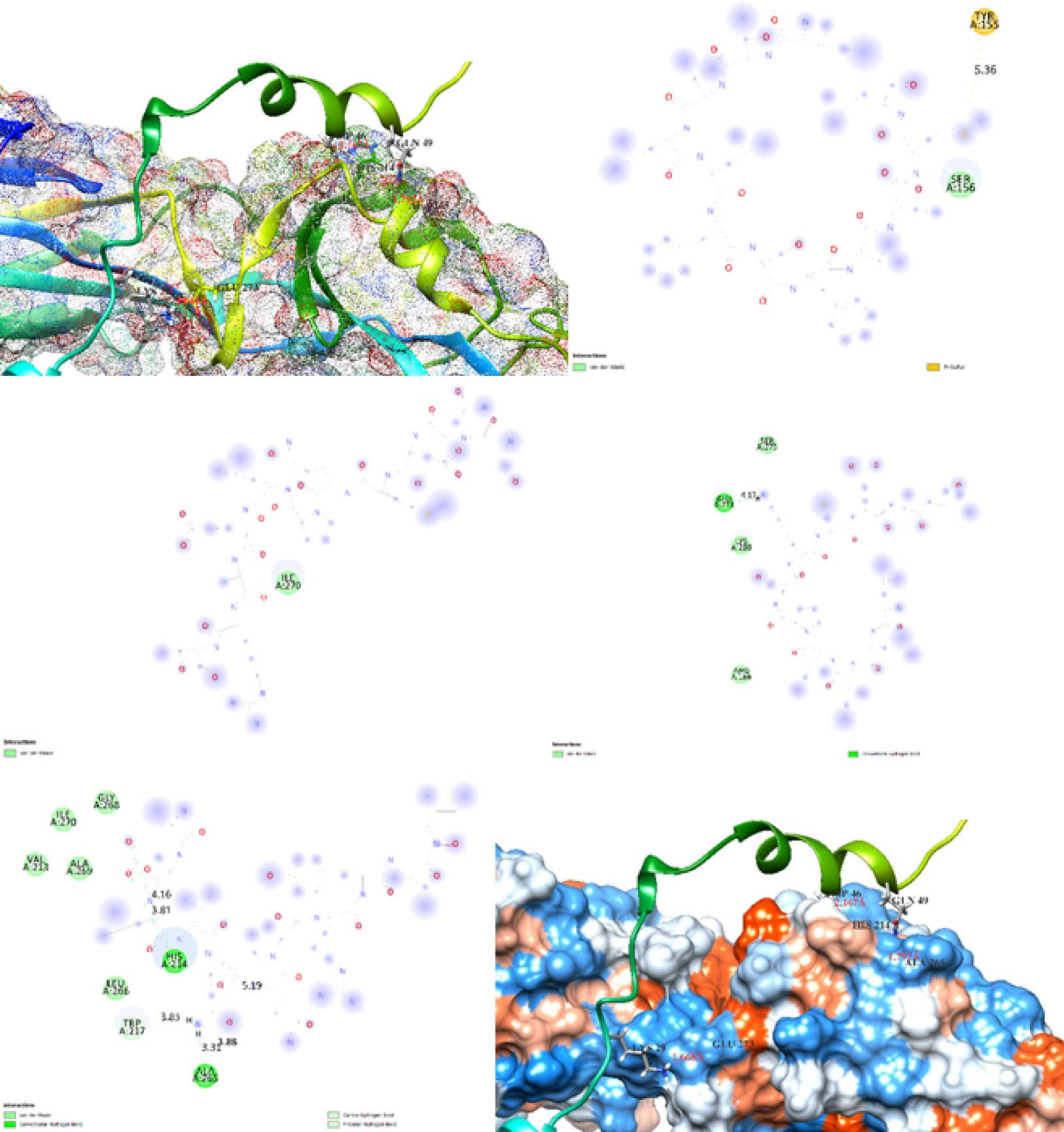
Representative 2D and 3D molecular docking models of Secapin-2 interaction with the West Nile virus (WNV) envelope glycoprotein. The predicted ligand–protein complexes illustrate hydrogen bonding and hydrophobic interactions between Secapin-2 and amino acid residues within the binding pocket of the WNV envelope protein. Secapin-2 is represented in green and gray, while interacting residues of the WNV envelope glycoprotein are shown in yellow and blue. The docking complex exhibited a binding free energy of − 5.1 kcal/mol.

Based on the observed in vitro inhibitory activity of the tested compounds against WNV, an in silico analysis was conducted to explore the differential binding modes of the ligands with the WNV envelope glycoprotein. Molecular docking was complemented by MD trajectory analysis to further evaluate the interactions between melittin and the target protein, including docking scores and hydrogen-bonding patterns. The results demonstrated strong and stable interactions with key active-site residues of the WNV envelope glycoprotein (Figs. 7, 8, 9, 10 and 11; Table 2).

**Table 2:** Docking scores and hydrogen bonding WNVenvelopeglycoprotein(PDB ID: 2i69) within bee venom components.

Free binding energy of the ligands, temperature (T) = 298.15 K					
Bee venom peptides	WNV envelope glycoprotein				
Apamin	–5.2 kcal/mol				
	Donor SER 341 LEU 355 LEU 355 ASN 359 ASN 2 CYS 3 ARG 13 ARG 13 ARG 13 ARG 13 HIS 18 HIS 18	Acceptor ARG 13 HIS 18 HIS 18 GLN 16 THR 300 ASN 359 SER 341 GLY 387 PRO 339 SER 341 ASP 37 ASP 37	Hydrogen SER 341 LEU 355 LEU 355 ASN 359 ASN 2 CYS 3 ARG 13 ARG 13 ARG 13 ARG 13 HIS 18 HIS 18	D. Å dist 2.572 3.453 3.187 2.874 2.902 3.100 2.908 3.010 2.924 2.950 2.755 2.804	D-H. Å dist 1.616 2.498 2.264 2.038 2.321 1.970 1.980 2.083 1.972 2.040 1.836 1.894
Tertiapin	–4.5 kcal/mol				
	Donor THR 148 LYS 298 THR 366 LYS 370 ASN 4 ARG 7 ARG 7 ARG 7 LYS 16 LYS 20	Acceptor CYS 18 ASN 6 LYS 20 GLY 19 GLY 160 THR 162 GLU 182 THR 162 GLU 150 LYS 370	Hydrogen THR 148 LYS 298 THR 366 LYS 370 ASN 4 ARG 7 ARG 7 ARG 7 LYS 16 LYS 20	D.Ådist 2.671 2.713 3.025 2.783 2.761 2.958 2.722 2.931 2.992 2.782	D-H.Ådist 1.735 1.832 2.480 1.795 1.845 2.055 1.889 2.000 2.279 1.763
Melittin	–13.0 kcal/mol				
	Donor GLU 273 TRP 19 ARG 22 ARG 22	Acceptor GLN 25 ASP 28 MET 46 ASN 47	Hydrogen GLU 273 TRP 19 ARG 22 ARG 22	D. Å dist 2.917 2.779 2.937 2.799	D-H. Å dist 2.162 1.814 1.969 1.868
Mast cell degranulating peptide	–10.8 kcal/mol				
	Donor GLN 264 SER 3 TYR 20 HIS 35	Acceptor PHE 21 GLU 244 TRP 217 GLU 26	Hydrogen GLN 264 SER 3 TYR 20 HIS 35	D. Å dist 2.882 2.544 3.114 2.681	D-H. Å dist 1.905 1.554 2.386 1.796
Secapinpeptide	–5.1 kcal/mol				
	Donor HIS 214 LYS 29 GLN 49	Acceptor ASP 46 GLU 273 ALA 265	Hydrogen HIS 214 LYS 29 GLN 49	D. Å dist 3.102 2.690 2.804	D-H. Å dist 2.167 1.668 1.792

## Discussion

WNV is an emerging viral pathogen with outbreaks of varying severity reported worldwide^2^. In recent years, the number of infections has continued to rise, yet no clinically approved vaccines or effective antiviral drugs are available for human treatment^4^. Although progress in antiviral drug development has accelerated- particularly over the past two decades, which have been among the most active periods in the field- therapeutic options for WNV remain limited^33^. Moreover, the high cost of antiviral medications underscores the urgent need for effective preventive measures^34^. Lately, the therapeutic and prophylactic potential of bee venom (BV) has attracted considerable scientific interest^35^. Studies have demonstrated its effectiveness against various cancer types as well as its notable antiviral activity^14^; Atwa et al.^35^. In vivo studies have further demonstrated the antiviral potential of BV and its constituents. Uddin^36^ showed that non-cytotoxic concentrations of BV and its main peptide, melittin (MEL), significantly suppressed the replication of various enveloped viruses including Influenza A virus (PR8), Vesicular Stomatitis Virus (VSV), Respiratory Syncytial Virus (RSV), and Herpes Simplex Virus (HSV) as well as non-enveloped viruses such as Enterovirus-71 (EV-71) and Coxsackie Virus (H3) when tested in rats. More recently, Sleman et al.^37^ reviewed the broad-spectrumantiviral property of HBV against numerous viruses, such as influenza viruses, herpes simplex virus, HIV, hepatitis viruses, and emerging coronaviruses. Also, Sonje et al.^38^ reviewed that BV contains over 40 bioactive compounds, with melittin comprising approximately 50% of the venomʼs dry weight and demonstrating significant antiviral, antibacterial, and anticancer effects.

So, the current study is an attempt to evaluate the crude BV and its nanoemulsion(NE) form on WNV. The results indicated that NE could deliver BV as more effective and enhanced its physical characterization; this finding is compatible with Yousefpoor et al.^23^. The dynamic light scattering (DLS) revealed that the size of bee venom nanoemulsion was greater than that of the crude BV. The nanoemulsion exhibited a slightly lower PDI than crude BV, indicating a comparatively narrower particle-size distribution indicating that the NE was more stable and more effective in biological behavior. These results are in line with those reported by Taher et al.^39^, who found that the DLS analysis revealed that the size of nanoparticles of BV with chitosan increased with increasing chitosan concentration and their PDI was less than 0.5.Yousefpoor et al.^23^ investigated the ability of BV and its nanoemulsion (NE) formulation to penetrate rat skin, noting that interactions between NE components and the molecular structure of BV may facilitate this permeability. Their results demonstrated that the NE was capable of delivering BV across the skin layers. In addition, dynamic light scattering (DLS) characterization showed that the particle size and polydispersity index (PDI) of the BV-NE increased as the BV concentration increased. Conversely, increasing the surfactant content led to a reduction in particle size, consistent with previous observations by Khani et al.^40^. This reduction is likely due to the higher number of surfactant molecules stabilizing the droplets and enabling the formation of smaller, more uniform nanoemulsions^41^.

Zeta potential plays an important role in characterizing colloids and nanoparticles in suspension, as it influences particle interactions and colloidal behavior^42^. In the present study, the bee venom nanoemulsion exhibited a zeta potential of approximately + 23.0 mV, while crude bee venom showed a value of − 23.7 mV. These values indicate moderate electrostatic stabilization but do not alone provide sufficient evidence for strong colloidal stability. Therefore, the interpretation of nanoemulsion stability was based on the combined evaluation of zeta potential together with the time-resolved DLS analysis, which demonstrated only minor changes in particle size and PDI during the 30-day storage period.

The observed physical stability of the nanoemulsion may also be attributed to steric stabilization provided by the emulsifying components, in addition to electrostatic effects. Similar observations were reported by Gan and Wang^43^ and Taher et al.^39^, who attributed reductions in zeta potential to interactions between venom components and carrier materials, including adsorption of venom molecules onto the particle surface. In another study, El Didamony et al.^44^ reported zeta potential values of 50.3 mV for chitosan nanoparticles and 44.6 mV for BV-loaded nanoparticles.

Considering the characteristic features of crude BV and nanoemulsion bee venom and testing them on WNV, we found that the nanoemulsion bee venom gave more positive results than the crude BV, being it was more effective antiviral compared to the crude, and it was stronger and better than the bee venom in the Vero cells. The results were confirmed by RT-qPCR, and the nanoemulsion achieved antiviral activity at a substantially lower non-cytotoxic concentration compared with crude BV, using 0.39 µg/ml for the nanoemulsion and 10 µg/ml for the crude form. This is considered preferable, economical, affordable, and effective. Previous studies by Ramadan et al.^45^ and Yousefpoor et al.^24^ evaluated the effect of crude bee venom (BV) on WNV and demonstrated that BV has a significant antiviral effect. In the present study, the choice of cell line may have influenced the antiviral activity of BV, as the percentage reduction achieved by crude BV was lower than that of the nanoemulsion formulation. This observation aligns with the findings of Ramadan et al.^45^, who reported that co-incubation of Vero cells with BV prior to WNV infection did not inhibit viral replication. In contrast, Alkhalefa et al.^46^ tested BV against Foot-and-Mouth Disease Virus (FMDV) in Baby Hamster Kidney-21 (BHK-21) cells and found a significant reduction in viral titers (25.7%) when the virus was treated directly with BV, indicating antiviral effect. Furthermore, BV treatment of cells prior to infection reduced viral titers by 20.8%, suggesting an induced antiviral state within the cells.

Although the efficiency of crude BV is less than NE in Vero cells, this does not reduce the efficiency of BV and its components against WNV. Therefore, to confirm the effectiveness of BV against WNV, molecular docking was applied between the components of BV (apamin, tertiapin, melittin, mast cell degranulating peptide, secapin-2peptide) on the WNV envelope glycoprotein and the docking scores were–5.2, − 4.5, − 13.0, − 10.8, − 5.1 kcal/mol, respectively. According to the results of this study, it was shown that BV has a very strong effect and can attack the virus in its inhibition sites by breaking hydrogen bond of virus’s protein.Protein–protein and protein–peptide interactions are essential for understanding the structural and functional properties of biomolecular complexes. Molecular docking has emerged as a powerful computational approach for characterizing these interactions and predicting binding behaviors among diverse biomolecules^47^. Despite docking analysis reflects the strongest predicted interaction, experimental interaction needs validation before a definitive antiviral mechanism can be established. The WNV envelope glycoprotein was selected because of its essential role in viral attachment and entry into host cells. Although NS3 protease and NS5 polymerase are also established antiviral targets, evaluation of bee venom peptide interactions with these proteins was beyond the scope of the present study and warrants future investigation. In docking analysis, the best-docked pose, typically the one with the lowest glide score, reflects the strongest predicted interaction^25^; Abd El^48^. To date, no studies have assessed protein–peptide docking between bee venom (BV) components and WNV proteins. However, Mustafa et al.^49^ investigated interactions between BV peptides and the DNA-directed RNA polymerase of viruses in the *Capripoxvirus* genus (family Poxviridae). Their results showed that the peptides melittin and secapin-1 achieved the lowest binding energies (− 106.9 ± 7.2 kcal/mol and − 101.4 ± 11.3 kcal/mol, respectively) and formed highly stable complexes. Molecular dynamics simulations further supported the stability of the melittin-polymerase complex, indicating sustained peptide-protein binding throughout the simulation period. Similarly, Muzammal et al.^50^ reported that phospholipase A2 from honeybee venom may serve as a potential antiviral agent against the Ebola virus. Additional computational analyses of hymenopteran venoms have also shown favorable binding of venom-derived compounds within the active pocket of the SARS-CoV-2 3CL protease^25^; Abd El^48^.

The main limitations associated with BV include dose-related cytotoxicity and mild allergic responses, with rare instances (< 0.1%) of anaphylaxis. Overall, the antiviral potential of BV is strongly supported by preclinical studies; however, available clinical evidence in humans remains limited^37,38^. Safety concerns, including allergic reactions and challenges related to formulation standardization, also require careful consideration^38^.

In addition, the present study did not include plaque reduction assays or time-of-addition experiments (pre-, co-, or post-treatment). Therefore, the precise antiviral mechanism of crude bee venom and its nanoemulsion against WNV, including possible virucidal, viral entry inhibitory, or replication inhibitory effects, could not be conclusively determined. Furthermore, antiviral comparisons were performed using the respective maximum non-cytotoxic concentrations rather than equivalent bee venom doses, and antiviral activity was evaluated only at these concentrations. Consequently, EC₅₀ and Selectivity Index (SI) values could not be determined. Further mechanistic, dose-matched, and in vivo studies are warranted to better characterize the antiviral mode of action and therapeutic potential of these formulations.

In conclusion, BV-NE demonstrated promising preliminary in vitro anti-WNV activity; however, further mechanistic, toxicological, and in vivo investigations are required to validate its therapeutic potential. Future studies evaluating melittin-based monotherapy and combination therapeutic approaches are also recommended. In addition, because the antiviral assays in the present study were performed exclusively in Vero cells, which lack a functional interferon response, further validation using human and mosquito-derived cell models is warranted to better characterize the observed antiviral effects.

## Data Availability

The datasets generated and/or analyzed during the current study are publicly available in the Zenodo repository. The data can be accessed through Zenodo Record No. 20849245 at https://zenodo.org/records/20849245. [https://doi.org/10.5281/zenodo.20849245].
